# Narrative Exposure Therapy versus treatment as usual in a sample of trauma survivors who live under ongoing threat of violence in Rio de Janeiro, Brazil: study protocol for a randomised controlled trial

**DOI:** 10.1186/s13063-021-05082-2

**Published:** 2021-02-26

**Authors:** Fernanda Serpeloni, Jeanine Arabella Narrog, Simone Gonçalves de Assis, Joviana Quintes Avanci, Samuel Carleial, Anke Koebach

**Affiliations:** 1 Department of Studies on Violence and Health Jorge Careli, National School of Public Health, Oswaldo Cruz Foundation, Avenida Brasil 4036, 700 Manguinhos, Rio de Janeiro, 21040-361 Brazil; 2 Vivo International e.V., Postbox 5108, 78430 Konstanz, Germany; 3 Department of Psychology, University of Konstanz, Universitätsstraße 10, Konstanz, 78464 Germany

**Keywords:** Narrative Exposure Therapy, PTSD, Trauma, Depression, Community violence, Violence

## Abstract

**Background:**

One in three individuals who live in Rio de Janeiro experience a traumatic event within a period of 12 months. In the *favelas* particularly, trauma exposure is ongoing. Psychological sequalae include posttraumatic stress disorder (PTSD), depression and other mental disorders. Trauma-focused therapy approaches have emerged as the treatment of choice when the dangerous events are over, but symptoms have remained for an extended time period. Ideally, the victim is in a safe context during treatment. However, frequently, survivors cannot escape from situations characterised by ongoing threat and traumatic stress. The aim of this study is to research the effectiveness of Narrative Exposure Therapy in a sample of PTSD patients living under these conditions.

**Methods:**

Individuals fulfilling the criteria for PTSD and who live in conditions of ongoing community violence (i.e. in the *favelas*) in Rio de Janeiro will be randomly assigned to one of two treatments: Narrative Exposure Therapy (NET) or treatment as usual (TAU). Clinical endpoints will be primarily PTSD and secondarily symptoms of shutdown dissociation, depression, substance involvement  and functionality.

**Discussion:**

Effective treatment for PTSD patients who live in unsafe conditions could substantially reduce suffering of individuals and their families in Brazil. Based on this result, the extent to which such interventions may be useful as a first step in tackling the consequences of violence on a global scale will be discussed.

**Trial registration:**

Deutsches Register Klinischer Studien (German Clinical Trials Register) DRKS00017843. Registered on September 24, 2019

**Supplementary Information:**

The online version contains supplementary material available at 10.1186/s13063-021-05082-2.

## Administrative information

**Table:** 

**Data category**	**Information**
Title {1}	Narrative Exposure Therapy versus treatment as usual in a sample of trauma survivors who live under ongoing threat of violence in Rio de Janeiro, Brazil: study protocol for a randomised controlled trial
Primary registry and trial identifying number {2a and 2b}	Deutsches Register Klinischer Studien (German Clinical Trials Register) Title: Effect of Narrative Exposure Therapy versus treatment as usual in a sample of trauma survivors who live under ongoing threat: a randomised controlled trial in Rio de Janeiro, Brazil German Clinical Trials Register (Deutsches Register für klinische Studien; drks.de) DRKS00017843; September 24, 2019
Protocol version {3}	January 16, 2021 (Version 1.1)
Funding {4}	Young Scholar Fund of the University of Konstanz, Universitätsstraße 10, 78,464 Konstanz, Germany and vivo international e.V.
Author details {5a}	**Roles and responsibilities:** Fernanda Serpeloni, Dr rer nat, MA Psych, co-investigator & leading field coordinator (1, 2) Jeanine Arabella Narrog, MA Psych, clinical supervisor (NET) (2) Simone Gonçalves de Assis, Prof Dr rer soc, scientific and local supervisor (1) Joviana Quintes Avanci, Prof Dr rer soc, field coordinator and local supervisor (1) Samuel Carleial, Dr rer nat, MA Biol, data analyst (3) Anke Koebach, Dr rer nat, MA Psych, principal investigator (2, 3) (1) Department of Studies on Violence and Health Jorge Careli, National School of Public Health, Oswaldo Cruz Foundation, Avenida Brasil 4036, 700 Manguinhos, Rio de Janeiro, 21040-361, Brazil (2) Vivo International e.V., Postbox 5108, 78430, Konstanz, Germany (3) Department of Psychology, University of Konstanz, Universitätsstraße 10, Konstanz, 78464, Germany
Name and contact information for trial sponsor {5b}	Dr Anke Koebach, University of Konstanz, Universitätsstraße 10, 78464 Konstanz, Germany, E-Mail: Anke.Koebach@uni-konstanz.de
Role of sponsor {5c}	The sponsor-investigator is responsible for study design, data collection, management, analysis, and interpretation of data; writing of the report; and the decision to submit the report for publication.

## Introduction

### Background and rationale {6a}

About 114 million citizens of Brazil live in subnormal agglomerates (or *favelas*) [1]. These districts are characterised by social vulnerabilities such as lack of governmental services, poverty and high levels of violence [2]. About 35% of deaths by external causes in Brazil have been classified as homicides [3, 4]. In Rio de Janeiro particularly, the Western parts present with a high density of favelas. Here, the rate of homicides is about 10 times higher than in neighbourhoods of the South [5]. It is postulated that criminal organisations that are largely financed by trafficking of drugs and firearms play a major role in the escalation and the maintenance of high rates of violence [4]. The growing participation of children and youths is particularly alarming. The majority of individuals affected by urban violence are 25 years or younger [1]. According to a recent study by Ribeiro et al. [6], every third person in Rio de Janeiro had experienced a new traumatic event within 1 year. In the same sample, 87% reported at least one lifetime traumatic event [7]. Furthermore, insecurity and poverty affect familial relations and health [8]. In Brazil, the overall prevalence of intimate partner violence is estimated at about one third [9]. In a recent study, Serpeloni et al. [10] found that more than 50% of women who live in São Gonçalo city near Rio de Janeiro were exposed to intimate partner violence and 99% to community violence (e.g. witnessing fights, being threatened with a knife or a gun or witnessing killings). The risk of violence exposure remains high during pregnancy, a particularly sensitive period for mother and child. Common forms of violence are urban violence (60%), death of a close person (43%), accidents (34%), exposure to human remains (33%) or domestic violence (20%) [7]. About 71% of children reported hearing gunshots [7], 44% experience severe physical violence at home [11] and 22% of families experienced severe intimate partner physical violence [12]. In Brazilian women, mental health problems are particularly related to experiencing intimate partner violence [13]. Furthermore, family violence is a major determinant of mental health problems in children [12, 14]. Luz et al. [7] report an overall prevalence of 11% of posttraumatic stress disorder (PTSD) in Rio de Janeiro, assumedly with higher rates in low-income districts. Patients with PTSD have shown to be at a heightened risk for other mental disorders and also for experiencing more traumatic events. While subclinical tendencies of hypervigilance or avoidance may be adaptive at first, they substantially undermine the natural capacity to seek and avoid certain environments and intimate relationships. Evidence-based treatments for PTSD have been developed and tested [15]. Narrative Exposure Therapy (NET) [16] has emerged as an evidence-based treatment for patients in low- and middle-income countries and refugees, all of whom have been exposed to multiple traumatic stressors; for a review and meta-analysis, see Siehl et al. [17]. A substantial body of evidence shows the effectiveness of NET for patients who live in relatively safe conditions. Evidence is scarce however for circumstances of ongoing violence. Studies from post-conflict Eastern DR Congo with former combatants [18] and Iraqi women who live in a relationship with intimate partner violence [19] suggest beneficial but moderate effects so far.

Symptoms of posttraumatic stress are the result of intrinsic mnesic dynamics induced by the inherent properties of storing and retrieving information gathered during (multiple) traumatic events [20]. Fused neural fear associative networks of sensory, emotional, interoceptive and cognitive representations are separated from contextual and episodic memory systems [21]. Alarm responses can become activated by even small and subtle prompts. Re-experiencing occurs without the awareness of past and present. Intrusions, nightmares or flashbacks occur with a sense of the trauma happening again ‘here and now’. Trauma-focused therapy, in particular Narrative Exposure Therapy [16], is a specific form of psychotherapy for posttraumatic stress symptoms based on psychological theories of memory storage and retrieval [22]. During this treatment, the participant develops a narrative of traumatic events, which consolidates fragmented memories by connecting the associative representations with their respective context of time, place and situation (‘where’, ‘when’ and ‘what’). General trauma-related cues such as heart beating, sweating, fear, etc. are likely to occur in everyday life in a context of ongoing violence. Is it nevertheless possible to anchor the most traumatic events in the past and reduce symptom severity of PTSD? If so, is NET superior to treatment as usual and could improve care of trauma survivors?

### Objectives {7}

The aim of the study is to test the effectiveness of NET in a setting on ongoing violence in comparison to a treatment as usual (TAU) control group.

### Trial design {8}

The present study is a two-arm parallel group, observer-blinded, randomised, actively controlled clinical superiority trial with an allocation ratio of 1:1.

## Methods: participants, interventions and outcomes

### Study setting {9}

The trial is implemented in health centres in Rio de Janeiro (Brazil) in a joint project of the University of Konstanz and Claves/ENSP/Fiocruz. Collaborating health centres include Centro de Saúde Escola Germano Sinval Faria ENSP/Fiocruz, ENSP/Fiocruz (Manguinhos), Centro Municipal de Saúde Heitor Beltrão (Tijuca), potentially Clínica da Família Odaléa Firmo Dutra (Grajaú).

### Eligibility criteria {10}

Referred from the public health centres, individuals will be administered a baseline interview to be tested for eligibility. Eligibility criteria for participation in the trial are the following:

- ≥ 18 years
- Diagnosis of PTSD according to DSM-5
- Agreement to the written informed consent

Exclusion criteria:

- Neuropsychiatric symptoms
- Acute psychotic symptoms (and/or under neuroleptic treatment)
- Other neurological disorder(s)

### Who will take informed consent? {26a}

Information about the trial is provided by the health centre and later in more detail at the beginning of the baseline interview that is conducted by trained local interviewers. Informed consents include a description of the study and the procedures and potential benefits and risk. The participants are also provided with an explanation about their right to discontinue participation at any point, that the treatment will be free and that there will not be a financial benefit besides the remuneration of transport to treatment and interview sessions. The informed consent can be found in Additional file 1.

### Additional consent provisions for collection and use of participant data and biological specimens {26b}

Not applicable as no biological data specimen will be collected.

## Interventions

### Explanation for the choice of comparators {6b}

In spite of the well-documented effectiveness of NET in general, there is a lack of evidence in comparison with symptom alleviating techniques provided in established health structures in a context of ongoing violence. Symptom alleviating techniques are commonly used to treat trauma-related symptoms when trauma-focused treatment cannot be applied due to the lack of stability of the patient (and his/her environment).

### Intervention description {11a}

#### NET counsellors

Trained Brazilian health personnel (medical/family doctors, psychologists) and advanced psychology students will deliver NET under constant supervision by a NET expert.

#### Narrative Exposure Therapy

NET will be delivered in 8–12 individual sessions of 90–120 min each in length by Brazilian NET therapists. The flexible number of sessions allows to test NET under naturalistic conditions and to adapt to the client’s trauma history. The initial session involves the construction of a lifeline whereby traumatic events are mapped out in chronological order and situated in the context of the patients’ lifetime period, as well as the wider systemic, and socio-political context at the time. A therapy plan is constructed according to the idiosyncratic experiences of the individual and traumatic events. In the subsequent exposure sessions, the most traumatic experiences are re-lived (emotional, sensory, cognitive, interoceptive) in order to transform the fragmented report of traumatic events into a coherent narrative. The therapist makes a written record of the event, and at the beginning of the next session, the narration is read to the patient and corrected for potential discrepancies. In the last session, the participant receives a written report of his/her biography.

#### Treatment as usual

TAU receives (or continues to receive) the services of our collaborating health centre (where they have also been recruited from). The treatment is not standardised and typically targets the symptoms reported by the patient (often diagnosed as hypertension, depression, panic disorder). TAU includes pharmacotherapy, empathic listening, problem solving and practical or social support [23]. It does not include deliberate trauma exposure which is a key characteristic of evidence-based trauma therapy. Treatment doses will be assessed retrospectively during follow-up assessments.

### Criteria for discontinuing or modifying allocated interventions {11b}

Study participation will be discontinued if a patient withdraws their written informed consent. In case of a serious adverse event (SAE), we will investigate a causal link between intervention and the occurrence of SAE and evaluate whether trial continuation would be a risk to the client, and whether modifications should be made to the intervention (e.g. support sessions). SAEs are defined as events that (1) result in death, (2) are life-threatening, (3) require hospitalisation or cause prolongation of existing hospitalisation, (4) result in persistent or significant disability or incapability, (5) are a congenital anomaly or birth defect or (6) require intervention to prevent permanent impairment or damage (such as acute suicidality). All SAEs that are reported by the patients will be evaluated by the clinical psychologist on site and the PI. Whenever a patient is withdrawn from the trial, the reasons for the withdrawal or treatment discontinuation together with the corresponding dates will be recorded. If a patient completely drops out from the study, a final examination will be conducted if possible. In particular, every effort will be taken to assess the primary outcome at follow-up. If a patient does not return for a scheduled visit, every effort will be made to contact the person to arrange further visits according to the protocol. Depending on the number of dropouts, missing values in primary outcome may either be excluded from the analysis or replaced according to the intention-to-treat principle or multiple imputation methods, respectively.

We expect that our sample of patients living in districts with high levels of urban violence may experience new traumatic events or death of family members, relatives or friends during the period of treatment. Patients will remain in the trial, but exposure sessions may be paused for few weeks. Support sessions may be provided according to the patient’s needs.

### Strategies to improve adherence to interventions {11c}

Adherence to the NET manual will be ensured through the narrations produced in NET and close supervision by a NET expert (2 h per week) recording the outcome for the according sessions.

### Relevant concomitant care permitted or prohibited during the trial {11d}

In both arms, patients are permitted to continue psychopharmacological medication as prescribed by their psychiatrist (unless prescribed neuroleptics indicating acute psychotic symptoms).

### Provisions for post-trial care {30}

If one of the conditions is more effective, patients who are still need (fulfilling the diagnostic criteria for PTSD) will be offered the according treatment.

### Outcomes {12}

Outcomes will be measured 3 and 6 months after the treatment. PTSD symptoms will be assessed as primary outcome using the PTSD Symptom Scale Interview for DSM-5 [24]. The Patient Health Questionnaire [25] will be used to measure depression severity. To assess trauma-related dissociation, we will use the Shutdown Dissociation Scale [26]. Substance involvement will be evaluation with the Alcohol, Smoking and Substance Involvement Screening Test [27]. Functionality will be assessed with the Work and Social Adjustment Scale [28]. Ongoing violence will be assessed by the scale Things I’ve seen and heard [29] and three questions asking whether the client has experienced any threat to physical or social integrity or was actively involved in violent perpetration.

### Participant timeline {13}

An appointment for baseline assessment of trauma-related psychopathology will be scheduled by our study focal point with consenting individuals. Trained psychological interviewers will administer a structured, paper-based clinical interview (baseline, T0) to determine eligibility. Eligible patients will then be randomised and those allocated to the experimental condition will be contacted to start treatment at the next possible time point. Patients in the control condition will continue to benefit from the services of public health centres. Three and 6 months later, follow-up interviews (T1 and T2) will be conducted with the participants.

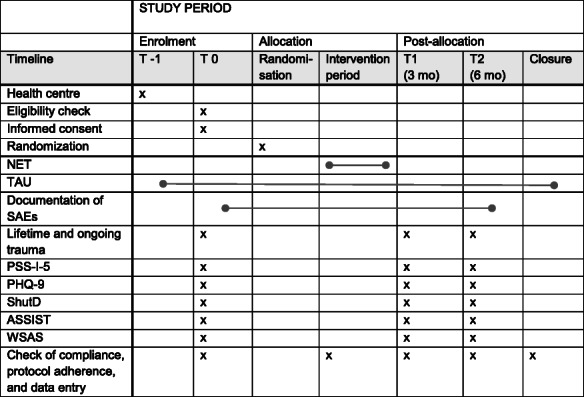



### Sample size {14}

Following similar studies in regions with ongoing violence, sample size per group was calculated based on a medium to high within-group Cohen’s *d* effect size for our primary outcome measure PTSD [18, 19, 30] with a power of >.70 and a significance level of .05, a sample size of 25 per group was suggested. However, to allow for a more robust analysis testing for a smaller effect due to the ongoing violence and in regard to covariates (level of ongoing violence) and secondary measures (depression, dissociation, substance involvement and medication, functioning), we will aim at a sample size of 30 per group.

### Recruitment {15}

Individuals are referred by the public health centres based on apparent trauma symptoms (pre-selection of eligible patients).

## Assignment of interventions: allocation

### Sequence generation {16a}

Eligible participants will be randomised to NET or TAU controlling for PTSD symptom severity and gender using a stratified block design for the initial group of 51 participants. For this, we will use the sample function in base R [31] setting a specific seed to allow for the results to be replicated. Participants who join the study later on will be randomised using a naïve minimization technique in Minimizer v.1.0.1 [32] which allows for a continuous randomisation of incrementing sample size. Minimization is based on techniques that allow to control for imbalance among patient factors and outcomes, balancing sample sizes per treatment groups [33].

### Concealment mechanism {16b}

Unpredictability of assignment will be ensured by implementing randomisation of participants after the baseline interview and by one of the authors who is not involved in the clinical work (SC, based in Konstanz, Germany).

### Implementation {16c}

Participants will be enrolled by the study coordinator (FS) and relevant information (participant code, sex and PTSD symptom severity) will be forwarded for randomisation (SC). Randomisation will consider sex (male or female) and PTSD symptom severity categories based on the PSS-I sum scores (low 7–25; low-medium 26–40; medium-high 41–55; and high 56–80).

## Assignment of interventions: blinding

### Who will be blinded {17a}

Trained master students in psychology will be blinded for the treatment condition of the participant, and they will conduct the baseline and follow-up interviews.

### Procedure for unblinding if needed {17b}

Not applicable as participants will be aware about whether they receive TAU or NET.

## Data collection and management

### Plans for assessment and collection of outcomes {18a}

PTSD symptoms will be assessed using the PTSD Symptom Scale Interview for DSM-5 (PSS-I) [24]. The PSS-I-5 assesses for PTSD diagnosis according to the DSM-5 [34]. Scores for each item range from 0 (not at all) to 4 (≤ 6 times a week/severe). Diagnosis will be ascertained according to the manual, and sum scores were derived by adding all items of clusters B-E (max 80). Participants will be instructed to answer in relation to an index trauma (dominant in intrusions) with 1-month time frame. The Patient Health Questionnaire (PHQ-9); [25]) will be used to measure depression severity. The nine-item questionnaire is to be scored from 0 (not at all) to 3 (nearly every day) according to the presence of the symptom in the previous two weeks. A sum score will be calculated for depression severity (max 27). To assess trauma-related dissociation, we will use the Shutdown Dissociation Scale (ShutD) [26]. This 13-item questionnaire assesses sensory deafferentation (e.g. transitory deafness or blindness), reduced nociception/analgesia, numbness, transitory paralysis, loss of speech/suppressed vocalisation, pseudo-neurological symptoms, (pre-) syncopes and out-of-body experiences on a 4-point scale (0—not at all to 3—several times a week). All items were added to a total score ranging from 0 to 39. Good psychometric properties were found in previous studies [35]. The Alcohol, Smoking and Substance Involvement Screening Test (ASSIST) [27, 36] will be applied to assess the risk of drug consumption for alcohol, cannabis and other recreational drugs; risk scores (‘low’, ‘moderate’ and ‘high’) indicate the probability of drug dependence as well as social, financial, health and legal problems consequent to the drug abuse. The Functionality will be assessed with the Work and Social Adjustment Scale (WSAS) [28]. Ongoing violence will be assessed by the scale Things I have seen and heard [29] and three additional questions asking whether the client has experienced any threat to physical or social integrity or was actively involved in violent perpetration. The applied instruments have successfully been used in various cultures with good psychometric outcomes (see e.g. [37] for PSSI-5; [38] and [39] for PHQ-9; [26] for ShutD; [36] for ASSIST; [40] for WSAS; [29] for Things I have seen and heard). Trained interviewers administer the questionnaires under close supervision in their first interviews until interviewing techniques and rating meets high standards. If possible, interviewers will do sit in to ensure interrater reliability.

### Plans to promote participant retention and complete follow-up {18b}

Follow-up data will be collected 3 and 6 months after treatment delivery. Patients who discontinued the trial will be provided care in the health centre.

### Data management {19}

Data will be stored and managed by Fiocruz during the active trial period (provision of treatments and follow up assessments) and transported to the University of Konstanz for later storage (10 years). To ensure data quality, entered questionnaires will be controlled. Before data analysis, we will also double check allocation of baseline and follow-up questionnaires via variables across time points and pursue further data quality checks (missings, invalid values, etc.). The data file will not include any information that would allow the identification of study participants. The final version of the data set will be shared via a private cloud server provided by the University of Konstanz which offers high security standards following e.g. ISO/IEC27001-2013 and offers a multi-layered encryption (e.g. via SSL/TLS, AES-256, end-to-end encryption).

### Confidentiality {27}

Pseudo-anonymisation, i.e. allocation of a code for each participant, is realised before the questionnaires are locked and archived in Fiocruz. A digital coding list combining the data secured with a 12-digit password is stored and updated regularly by the leading implementing personnel in charge. Questionnaires are entered in Fiocruz and missing values and inaccurate assessments are followed up. A data entry protocol is established and available upon request. Data integrity is further enforced before statistical analyses by checking validity of values and range.

### Plans for collection, laboratory evaluation and storage of biological specimens for genetic or molecular analysis in this trial/future use {33}

Not applicable as no biological specimen will be collected.

## Statistical methods

### Statistical methods for primary and secondary outcomes {20a}

To test the efficacy of NET in comparison to TAU in regard to primary (PTSD) and secondary (depression, shutdown dissociation, substance involvement, functionality) outcomes, we plan to use separate generalised linear mixed models (GLMMs) in R (R Core Team, 2018). To investigate the influence of ongoing violence, we will enter the sum scores for community and intimate partner violence as fixed model terms. To account for non-independence of measurements, we will include individuals as random model terms. To account for repeated measures, we will control the within-group variance for the effect of time, i.e. baseline and 3- and 6-month follow-ups, according to Field et al. [41]. To estimate the significance of the fixed terms, we plan to fit the models using maximum likelihood (ML) and compare models with and without each fixed term using likelihood-ratio tests (LRTs) [42]. In cases where interaction terms are significant, we will use post hoc Tukey tests using the R package emmeans [43] to assess which contrasts differ significantly. To correct for multiple comparisons controlling for the false discovery rate, we will adjust *P* values using the R built-in function p.adjust according to Benjamini and Hochberg [44]. Additionally, effect sizes will be estimated by calculating Cohen’s *d* [45] for within- and between-group differences. Clinically significant change will be calculated using the reliable change index, following Jacobson et al. [46] for clinical outcomes (PTSD, depression).

### Interim analyses {21b}

No interim analyses will be conducted.

### Methods for additional analyses (e.g. subgroup analyses) {20b}

Subgroup analysis might be considered for sex groups, level of violence exposure and/or perpetration.

### Methods in analysis to handle protocol non-adherence and any statistical methods to handle missing data {20c}

All participants who provide reliable data at baseline will be recorded in the dataset and analysed as randomised. Furthermore, patients who die before the evaluation of efficacy outcomes (truncation due to death) are part of the principal analysis incorporating the timing of death of the trial participant.

Every effort will be made to prevent missing data. One of the advantages of GLMM over ANOVA is that the number of observations is maximised by excluding missing values only, rather than removing complete cases with some or all missing values present. In addition, imputation methods implemented in the mice R package [47] will be considered if more than 10% of the values in the whole dataset are missing and not more than 30% per participant per assessment instrument.

### Plans to give access to the full protocol, participant-level data and statistical code {31c}

Data as well as statistical codes will be available for the research team and shared for replication purposes upon request. Researchers with significant scientific contributions to the article will gain authorship. Participants will be informed throughout the baseline interview about the possibility to obtain information on the study outcome at their local health centre. A layman-friendly text on the study results will therefore be provided at the latter when publishing the results. In order to allow access to the study results to researchers in low- and middle-income countries, we will prioritise publication in journals that offer open access.

## Oversight and monitoring

### Composition of the coordinating centre and trial steering committee {5d}

Fiocruz is responsible for the coordination of activities on site (e.g. arrangements with the health centres, ensure availability of rooms, etc.) and ensure study implementation follows the study protocol. Interviewer and NET trainings are led by AK and FS and supervision is conducted by FS, JN and AK. Fiocruz with the support of the University of Konstanz is responsible for data management, which encompasses all tasks concerning processing and utilisation of study data, with the aim of guaranteeing high-quality data and providing a valid study database for statistical analyses. The University of Konstanz (SC) will be responsible for conducting statistical analysis and the publishing of data.

### Composition of the data monitoring committee, its role and reporting structure {21a}

A data monitoring committee is not warranted due to the minimal risk of the interventions/high level of evidence from other settings.

### Adverse event reporting and harms {22}

We expect a low frequency of SAEs caused by the NET itself. However, given the hostility of the environment, SAE will most likely be frequent. SAEs are events that (1) result in death, (2) are life-threatening, (3) require hospitalisation or cause prolongation of existing hospitalisation, (4) result in persistent or significant disability or incapability, (5) are a congenital anomaly or birth defect or (6) require intervention to prevent permanent impairment or damage (such as acute suicidality). SAEs will be monitored by the NET counsellors and interviewers. Together with their supervisor, they will decide which cases have to be followed up as SAEs and reported to the supervising clinical psychologist on site and the PI.

### Frequency and plans for auditing trial conduct {23}

A continuously updated list prepared and followed up by the study coordinator (FS) with details that are not part of the assessment set or content of the treatment will provide information about relevant extraordinary events concerning trial conduct.

### Plans for communicating important protocol amendments to relevant parties (e.g. trial participants, ethical committees) {25}

Protocol modifications (e.g. changes to eligibility criteria, outcomes, analyses) will be reported to relevant parties (e.g. REC/IRBs, trial participants, trial registries, journals).

### Dissemination plans {31a}

Data as well as statistical codes will be available for the research team and shared for replication purposes upon request. Researchers with significant scientific contributions to the article will gain authorship. Participants will be informed throughout the baseline interview about the possibility to obtain information on the study outcome at their local health centre. A layman-friendly text on the study results will therefore be provided at the latter when publishing the results. In order to allow access to the study results to researchers in low- and middle-income countries, we will prioritise publication in journals that offer open access.

## Discussion

This study is conducted to contribute evidence on the efficacy of NET in the context of ongoing violence in comparison with a TAU control group. This is important because incidence of trauma-related disorders is highest in these settings and effective treatment most needed.

Trauma exposure is high in Brazil especially in the communities with high levels of crime such as the *favelas* in Rio de Janeiro, where individuals not only battle with poverty and lack of access to public health services, but also with drug trafficking organisations. At least 11% suffer the consequences of trauma at a clinical level [7]. Public health centres provide interventions that meet the basic needs of patients with physical illnesses but are not able to offer evidence-based trauma therapy. PTSD patients usually receive psychopharmacological medication for specific symptoms, which—with its side effects and potential for addictions—offers only a limited overall benefit [48, 49]. As well as causing significant suffering to the individual, the specific symptoms of PTSD (e.g. isolation, aggressiveness) alter societal dynamics towards pro-conflict violent behavioural chains that undermine the community’s peace and stability.

If NET with its properties of being a brief intervention with potential to be scaled up through non-mental health staff is found to be beneficial, new options will emerge to tackle trauma and violence in Brazil and postentially settings with ongoing violence in general. The extent to which the results will be generalisable to the whole country of Brazil and across the globe will be discussed.

Limitations of this study include the reliance on self-report of trauma histories and symptoms. These may be subject to bias of memory and also affected by social desirability, in both the control and intervention groups. Although adherence to the manual will be ensured through regular clinical supervision and written narratives of the session content, the absence of treatment fidelity ratings may be another limitation.

## Trial status

Interviewer and NET trainings have been provided successfully. Recruitment of patients, treatment delivery, and supervision are ongoing.

Protocol version 1.1 (last major updates September 29, 2019; last minor revisions February 4, 2020, and January 16, 2021 (plus amendment to the protocol)

Recruitment of the first patient: October 17, 2019

Last day of recruitment (expected): March 30, 2020, postponed to January 31, 2021

Last follow-up (expected): September 30, 2020, postponed to February 30, 2022

## Supplementary Information

**Additional file 1:** Model of informed consent.

## Abbreviations

PTSD: Posttraumatic stress disorder
NET: Narrative Exposure Therapy
TAU: Treatment as usual
RCT: Randomised controlled trial
DRKS: Deutsches Register Klinischer Studien (German Clinical Trials Register)
