# Supplementary material for: Narrative Exposure Therapy versus treatment as usual in a sample of trauma survivors who live under ongoing threat of violence in Rio de Janeiro, Brazil: study protocol for a randomised controlled trial
Source: Trials. 2021 Feb 26;22:165. doi: 10.1186/s13063-021-05082-2 (PMC7908771; doi:10.1186/s13063-021-05082-2)
Supplement: Supplementary file 1 — Additional file 1: Model of informed consent. [file 13063_2021_5082_MOESM1_ESM.pdf]

INFORMED CONSENT FORM

Dear participant,

You are being invited to participate in the "Narrative Exposure Therapy (NET) for post-traumatic stress treatment" project because you may or may not have experienced some life-long violence. The central objective of the study is to offer adequate and effective treatment to people who have experienced a situation of violence, integrating into the health services specialized therapeutic care for victims of violence who have developed post-traumatic stress reactions (PTSD). PTSD is characterised by difficulty in sleeping, difficulty in concentrating, intrusive memories of the traumatic event, places or people who remember the traumatic event, among others. All these reactions are normal after going through a very stressful event. However, if these symptoms persist for more than a month they can end up impairing performance at work and in family relationships. Talking about the trauma with the help of a specialized professional can help to relieve these symptoms.

To provide quality treatment it is necessary to check the effectiveness of Narrative Exposure Therapy (NET) to decrease PTSD symptoms compared to existing care at the health centre. The project is divided into two phases, the screening phase and the NET intervention phase. If you agree to participate, you will participate in phase 1, called the screening phase. In this phase you will be invited to respond to a structured interview. Questions will be asked about socio-emotional aspects, experiences of violence throughout your life and how you currently feel. Your participation in this phase 1 will not bring any direct benefit, however, you will be contributing to the research and knowledge of the effects caused by difficult life experiences. The information collected will help to better understand how people deal with stressful situations and the aspects that protect them throughout their lives. Regarding the indirect benefits, these interviews are part of a screening that may lead to participation in phase 2 of the project.

If you experience posttraumatic stress reactions, we will invite you to participate in the second phase of the study. In this phase the participants will be divided into two groups. One group will be assigned to the NET treatment and the second group will be assigned to the treatment that would already be offered in the health centre. The division into two groups is done randomly, that means it is done by a computer. After 3 and 6 months of therapy we will conduct follow-up interviews with all participants. All patients will be able to receive NET treatment after the study has been completed. NET is widely used in other countries and research shows its effectiveness, e.g. Germany, the United States, Sweden and Uganda. However, each person presents an individualized response, and for some the decrease in symptoms may be greater.

Rubrica Pesquisador:________

Rubrica Participante:________

If symptoms persist even after the NET intervention, you will continue to receive therapeutic follow-up at the Germano Sinval Faria School Health Centre (CSEGSF/Ensp) under the researchers' supervision.

The results should contribute to the development of more efficient intervention strategies and health promotion, thus aiming at improving the quality of life and well-being of your community and other regions around the world. The interview time is approximately one hour. The confidentiality and privacy of your information will be guaranteed. We will take all necessary care so that any data that may identify you is omitted. In this way, we will not disclose any data that could identify you.

According to research on traumatic experiences, talking about trauma and psychological suffering is an important opportunity for intervention and referral. If you are part of the NET care group, during the sessions you may feel strong emotions associated with the trauma, such as sadness and guilt. In addition you may feel sweating and shaking hands when talking about difficult events in life. All these emotions are part of the therapy sessions focused on trauma. Project therapists are trained to observe these reactions and intervene to promote relief and recovery of well-being as trained in Narrative Exposure Therapy (NET). The therapist will help associate these emotions with the traumas of the past, helping to develop the memory responsible for the context of events in their biographical history. In this way you will gain greater control over your emotional and physiological reactions. The most important thing is that you understand the step-by-step treatment so that you have control over what will happen during the sessions. If you have any questions about the procedure you can stop and ask. If you feel any emotion or physiological reaction, such as fear, heart beating faster, let the therapist know as this is part of the therapeutic process. The usual treatment received by the health centre refers to psychological care or the care provided by family doctors.

At any time during the research, or later, you can ask the researcher for information about participation and/or about the research, which can be done through the means of contact explained in this Term. During the interview possible emotional aspects associated with the traumatic experiences will be asked. In the event of any discomfort or embarrassment in relation to the questions asked, you have every right not to answer them. The researchers will conduct the interview with great sensitivity, as your well-being is of high priority to us. However, if you do not feel well after the interview, a psychologist from the research team will be available to talk to you. Your participation is voluntary. You will not have any expenses for the interview, which will be covered by the University of Konstanz (Germany). Nor will there be any form of payment for your participation.

The research material will be stored in a secure place in digital archives, but only the researcher and the research team delegated by the responsible researcher will have access to them. At the end of the research, all interview material will be kept on file for at least 5 years in accordance with national research regulations. You may have access to the results of the study, as well as the right to withdraw consent to the safekeeping and use of the stored research material at any time without any cost or prejudice, with withdrawal valid from the date of communication. For this purpose, you may contact the project coordinator through the contact information written in this term. A seminar will be held with the results for the residents of Manguinhos on the relationship between violence and post-traumatic stress disorder. The name of the participant won’t be revealed.

Rubrica Pesquisador:________ Rubrica Participante:________

You may decide to withdraw from the survey at any time without penalty of any kind. However, your participation is very important for the execution of the research. We therefore ask for your consent to participate in the survey in the terms mentioned above. This term is written in two copies, one for you and one for the researcher.

I declare that I agree to participate in the survey.
Name of the participant:_____________________________________
Signature:____
Date:____

Name of the interviewer:_____________________________________
Signature:____
Date:____

-contact info were included-
